# Antioxidant, Cytotoxic, and Antimicrobial Potential of Silver Nanoparticles Synthesized using *Tradescantia pallida* Extract

**DOI:** 10.3389/fbioe.2022.907551

**Published:** 2022-07-18

**Authors:** Irum Shahzadi, Syed Munawar Aziz Shah, Mohammad Maroof Shah, Tariq Ismail, Nighat Fatima, Maria Siddique, Ummara Waheed, Ayesha Baig, Aisha Ayaz

**Affiliations:** ^1^ Department of Biotechnology, COMSATS University Islamabad, Abbottabad Campus, Abbottabad, Pakistan; ^2^ Department of Pharmacy, COMSATS University Islamabad, Abbottabad Campus, Pakistan; ^3^ Department of Environmental Sciences, COMSATS University Islamabad, Abbottabad Campus, Abbottabad, Pakistan; ^4^ Institute of Plant Breeding and Biotechnology, Muhammad Nawaz Shareef University of Agriculture, Multan, Pakistan; ^5^ Combined Military Hospital, Abbottabad, Pakistan

**Keywords:** antioxidant, anticancer, antibacterial, antifungal, green synthesis, *Tradescantia pallida*

## Abstract

Silver nanoparticles have received much attention, due to their wide range of biological applications as an alternative therapy for disease conditions utilizing the nanobiotechnology domain for synthesis. The current study was performed to examine the antioxidant, anticancer, antibacterial, and antifungal potential of biosynthesized silver nanoparticles (TpAgNPs) using plant extract. The TpAgNPs were produced by reacting the *Tradescantia pallida* extract and AgNO_3_ solution in nine various concentration ratios subjected to bioactivities profiling. According to the current findings, plant extract comprising phenolics, flavonoids, and especially anthocyanins played a critical role in the production of TpAgNPs. UV–visible spectroscopy also validated the TpAgNP formation in the peak range of 401–441 nm. Further, the silver ion stabilization by phytochemicals, face-centered cubic structure, crystal size, and spherical morphology of TpAgNPs were analyzed by FTIR, XRD, and SEM. Among all TpAgNPs, the biosynthesized TpAgNP6 with a medium concentration ratio (5:10) and the plant extract had effective antioxidant potentials of 77.2 ± 1.0% and 45.1 ± 0.5% free radical scavenging activity, respectively. The cytotoxic activity of TpAgNP6 in comparison to plant extract for the rhabdomyosarcoma cell line was significantly the lowest with IC_50_ values of 81.5 ± 1.9 and 90.59 ± 1.6 μg/ml and cell viability % of 24.3 ± 1.62 and 27.4 ± 1.05, respectively. The antibacterial and antifungal results of TpAgNPs revealed significant improvement in comparison to plant extract, i.e., minimum inhibition concentration (MIC) 64 μg/ml against Gram-negative *Pseudomonas aeruginosa* while, in the case of antifungal assay, TpAgNP6 was active against *Candida parapsilosis*. These TpAgNPs play a crucial role in determining the therapeutic potential of *T. pallida* due to their biological efficacy.

## 1 Introduction

Green nanomaterials are a new and specialized field of nanoscience and nanobiotechnology. Nanobiotechnology is a combination of nanotechnology and biology in which nanosystems are utilized to help in the study of biological systems. Nanobiotechnology has a lot of potential uses, and researchers are increasingly interested in employing nanotechnology to increase therapy efficacy. Nanoparticles can be created to interact with the body at a molecular level, with a high level of functional specificity in this way (Emerich and Thanos, 2003; Fakruddin et al., 2012; Yaqoob et al., 2020). Nanomaterials have the potential to solve technical issues in therapeutics and biomedical science (Jiang et al., 2005; Shrivastava and Dash, 2009; Roostaee and Sheikhshoaie, 2020; Sharma and Hussain, 2020; Yaqoob et al., 2020). Metal-based nanoparticles like silver can enhance or reduce metabolic machinery (Jiang et al., 2005) and are also used as a tool to differentiate and detect biological alterations. Silver nanoparticles have received a lot of interest due to their unusual physical, chemical, and biological characteristics, as well as their potential applications in biology and medicine (Wijnhoven et al., 2009; Rai and Duran, 2011). Since ancient times, silver has been used as an antibacterial agent, and silver-based compounds are significantly less costly than gold-based compounds (Srikar et al., 2016). Furthermore, silver nanoparticles are non-toxic to eukaryotic cells like humans, whereas they are extremely poisonous to prokaryotic cells like bacteria, viruses, and fungi (Hussain et al., 2016). These characteristics of silver nanoparticles encourage the researchers to investigate its unique features in nanomedicine applications such as antimicrobial (Ahmed et al., 2016; Mohanta et al., 2017), antiplasmodial (Mishra et al., 2013), targeted drug distribution (Praveen et al., 2012; Lai et al., 2020), antifungal (Al-Otibi et al., 2021), anticancer (Castro-Aceituno et al., 2016; Sarkar and Kotteeswaran, 2018), antiplatelet (Krishnaraj and Berchmans, 2013), and wound healing (Rigo et al., 2013).

Physical and chemical procedures are not suited to produce silver nanoparticles due to low yields and the usage of hazardous chemicals. Bacteria, algae, fungi, and plants are among the biological substrates used to produce metallic nanoparticles, with plants gaining greater attention due to their superiority over other sources (Bagherzade et al., 2017). Plant-mediated nanoparticles are a new area of study that has gained a lot of biotechnological interest because of their various uses and the fact that, unlike microorganisms, they do not require complicated methods to preserve microbial cultures (Okaiyeto et al., 2021). Plants are also a preferred resource over microorganisms because of their widespread distribution and easy availability, as well as lack of gene mutation (Kavitha et al., 2013). As a result of these distinguishing characteristics, plant-mediated nanoparticle production has proven to be advantageous and promising in comparison to other approaches. In addition, multiple studies reported that biomolecules found in plant extracts, such as saponins, flavonoids, terpenoids, phenolics, alkaloids, and proteins, play a vital role in metal ion reduction and serve as stabilizing factors for nanoparticle synthesis (Mandal et al., 2005; Mikhailova, 2020). The green method of nanoparticle manufacturing has low to no toxicity, is environment-friendly, and is cost-effective, and a variety of plants and their extracts have been reported to be used in the process (Gour and Jain, 2019; Jagessar, 2020). Green biosynthetic protocols for the preparation of nanomaterials are the known trend in the 21st century among scientists. It is beneficial to use medicinal plants since their medicinal characteristics are added to the nanoparticles throughout the production process (Jagessar, 2020). Plant phytochemicals contain antioxidant properties, and plants with antioxidant properties contribute additional antioxidant, anticancerous, and antimicrobial properties to nanoparticles (Pasupuleti et al., 2013; Gour and Jain, 2019; Ji et al., 2021; Liu et al., 2022). Additionally, as reducing or capping agents, these phytochemicals play an important role in nanoparticle production.

*T*radescantia *pallida* belongs to the family Commelinaceae, also called purple queen. It is an ornamental plant and exhibits several important traditional medicinal properties. Hence, it is conventionally utilized as anti-inflammatory and antitoxic for improving blood circulation (Li, 2006). Previous studies of phytochemical analysis show that *T. pallida* contains medicinally significant compounds such as phenolics, flavonoids, tannins, alkaloids, and saponin (Huq et al., 2016) and is also a rich source of naturally colored compounds, especially anthocyanins (Jabli, 2018). These compounds of *T. pallida* extract reveal a great degree of effectiveness in antioxidant, antibacterial (Tan et, 2014), antitumor, cytotoxic, *in vivo* analgesic, thrombolytic, and membrane-stabilizing activities (Huq et al., 2016; Dash et al., 2020). The silver nanoparticles from the extract of *T. pallida* have been synthesized (Hasnain et al., 2019) but, to the best of our knowledge, no study dealing with the bio-guided synthesis of silver nanoparticles is reported in the literature for *T. pallida*.

Keeping in view all the effective properties of *T. pallida*, the current work was designed for the first time to show that silver nanoparticles from the aerial parts of *T. pallida* have antioxidant, cytotoxic, and antimicrobial properties. Therefore, in this investigation, *T. pallida* extract was utilized to synthesize silver nanoparticles from AgNO_3_ precursors, which were then characterized using various methodologies.

## 2 Materials and Methods

### 2.1 Plant Collection, Identification, and Drying

*T. pallida* aerial parts were collected in April and May 2018 from COMSATS University Islamabad nursery garden, Abbottabad Campus, Pakistan, and the plant was identified by a taxonomist. Dirt from sample plants was removed by washing with distilled water and the plants were dried in shade at 25°C. Further, the dried sample plants were ground using a mechanical grinder machine.

### 2.2 Plant Extract Preparation

*T. pallida* powder, 25 g, was mixed with 300 ml of 80% methanol (1:4; H_2_O:CH_3_OH v/v with 0.5% formic acid). Ultrasound-assisted extraction was conducted in a water bath with sonication and the procedure was repeated thrice, with the frequency set at 40 kHz and power at 300 W. The temperature of the mixture was maintained at 25 ± 5°C and the extraction time was kept at 1 h. The sample was sieved with Whatman filter paper, and the filtrate was dried in a rotary evaporator at 28°C (Ghafoor et al., 2009). The semisolid extract was further freeze-dried to evaporate moisture under vacuum and stored at 4°C until further use.

### 2.3 Quantitative Phytoconstituent Analysis

#### 2.3.1 Determination of Total Phenolic Content

The total phenolic content was estimated by the Folin–Ciocalteu method (Ainsworth and Gillespie, 2007). A mixture of 200 µl plant extract (1 mg/ml) and 1.5 ml 10-fold-diluted Folin–Ciocalteu reagent was kept at room temperature for 5 min and lastly, 1.5 ml of 6% sodium carbonate was added. The whole mixture was incubated at room temperature for 90 min. A spectrophotometer was used to measure the absorbance at 725 nm of all samples and the blank holding all the reagents excluding the extract. Gallic acid was used as standard.

#### 2.3.2 Determination of Total Flavonoid Content

The total flavonoid content was estimated by the colorimetric method (Pontis et al., 2014). The reaction mixture contained 1 ml plant extract (1 mg/ml), 4 ml dH_2_O, and 0.3 ml sodium nitrite (5%). Then 0.3 ml aluminum chloride (10%) and 2 ml NaOH (1 M) were added after 5 min. The volume was made up to 6 ml with distilled water. The absorbance was measured at 510 nm using a spectrophotometer against a blank containing all the reagents except the extract. Rutin was used as standard.

#### 2.3.3 Determination of Total Anthocyanin Content

The total anthocyanin content was estimated by a pH-differential method (Giusti and Wrolstad, 2001). Two buffer systems were used, i.e., KCl buffer (pH 1) and sodium acetate buffer (pH 4.5). One milliliter of extract (1 mg/ml) in different solvents was diluted with the corresponding buffer up to a volume of 6 ml. Absorbances of the dilutions were read at 520 and 700 nm. The total anthocyanins were measured as cyanidin-3-glucoside (C3G) equivalents.

### 2.4 Biosynthesis of Silver Nanoparticles

The silver nanoparticles were synthesized by reacting the extract of *T. pallida* and AgNO_3_ aqueous solution in various concentrations. The three concentrations (1, 5, and 10 mM) of AgNO_3_ and three concentrations (1, 5, and 10 mg/ml) of plant extract were used to prepare nine sets (1:1, 1:5, 1:10, 5:1, 5:5, 5:10, 10:1, 1:5, 10:10) of samples. Each reaction ratio mixture contained 50 ml aqueous AgNO_3_ and 50 ml methanolic plant extract from all nine sets of sample concentrations. These various concentrations were used to determine the effect of silver ion (Ag^+^) and plant extract on the synthesis of NPs. The reaction mixture of each sample was kept for 24 h at room temperature in dark for the reduction of silver ion (Ag^+^) into TpAgNPs. The presence of silver nanoparticles was determined by color change of the reaction mixture after incubation. The TpAgNPs solution was then centrifuged for 15 min at 10,000 rpm. The pallet of nanoparticles was obtained, and to remove undesired impurities the pallet was further washed three times with deionized water (Ashraf et al., 2020).

### 2.5 Biological Assays of Plant Extract and Biosynthesized TpAgNPs

#### 2.5.1 Radical Scavenging Activity

The antioxidant activity of the plant extract and TpAgNPs was estimated by DPPH (1,1-diphenyl-2-picrylhydrazyl) radical scavenging activity method (Liyana-Pathiranan and Shahidi, 2005). Two milliliters of DPPH (0.1 mM in methanol) was added in 1 ml (0.1 mg/ml) of extract and TpAgNPs. The reaction mixture was vortexed and incubated at room temperature for 45 min. The falcon tubes were protected with aluminum foil to avoid light contact and for better scavenging activity of DPPH. The decolorization of DPPH was determined at 517 nm with a blank containing DPPH solution without plant extract and TpAgNPs. Ascorbic acid was used as standard, and the percentage of radical scavenging activity was calculated. An analysis of linear regression was implemented to conclude the value of inhibitory control (IC_50_) for the sample.

#### 2.5.2 Cytotoxicity Assay

The anticancer potential of the extract and TpAgNPs was estimated by the MTT [3-(4,5-dimethylthiazol-2yL)-2,5-Diphenyl] test using rhabdomyosarcoma cell line (RDATCC# CCL-136) (Mativandlela et al., 2006). The cell culture media contained Dulbecco’s Modified Eagle Medium (DMEM; sterile filtered DMEM: (–)l-leucine, 4.0 mM l-glutamine, (–)l-methionine with 4.5 g/l glucose, phenol red, and sodium pyruvate). The suspension of the cell was made, 0.2 ml was applied to each well of the 96-well plates with a density of 1 × 10^5^ cells/well, and the plates were kept at 37°C in 5% CO_2_. After incubation of the plates for 2–3 days in 5% CO_2_ at 37°C, the medium was changed to a new DMEM and various samples (50 ml), and the plates were re-incubated for 2–3 days in 5% CO_2_ at 37°C. The experiment was then split into two. Following incubation, the first plate was treated for medium elimination and washed three times with PBS. MTT solution was administered in all the wells, and further, the plate was incubated in 5% CO_2_ at 37°C for 200–250 min. The extracting solution was added after 45 s of incubation, and the results were measured using a microplate reader at 570 nm. The cell viability percentage was calculated by the following formula:

Cell Viability% =(Mean OD/Control OD)∗100.

The samples were further studied for IC_50_.

#### 2.5.3 Antibacterial Assay

The Gram-negative strain of American Type of Culture Collection (ATCC) *Pseudomonas aeruginosa* (ATCC#15442) and Gram-positive strains of *Staphylococcus aureus* (ATCC 6538) and methicillin-resistant *S. aureus* (MRSA) were used to analyze the antibacterial activities of the plant extract and biosynthesized TpAgNPs.

Nutrient broth medium (Oxoid, England) was prepared for bacterial growth and autoclaved at 121°C for 15 min. The 24-h-old culture was used for inoculum preparation from a selected bacterial strain in nutrient broth. The turbidity of bacterial cultures was analyzed by comparing the McFarland turbidity standard (0.5 BaSO_4_). The nutrient agar (Britanialab) plating medium was made according to the manufacturer’s advice in distilled water and autoclaved for 20 min at 121°C. Petri plates were prepared by pouring 25 ml of nutrient agar near the flame and allowing to solidify.

To analyze the antibacterial activity of the plant extract and biosynthesized TpAgNPs, the agar well diffusion method was used (Shahzadi and Shah, 2015). The selected bacterial cultures were inoculated individually on Petri plates. A 6-mm sterile metallic borer was used for well preparation at an equal distance of 40 mm from each other and sealed. A volume of 5 mg/ml of plant extract and TpAgNP samples were dissolved in dimethyl sulfoxide (DMSO; Sigma-Aldrich, United States). Pure DMSO was used as negative control and 0.5 mg/ml ciprofloxacin (Sigma-Aldrich) antibiotic was used as positive control. To each well, 40 µl of sample (200 µg), positive control (20 µg), and negative control (DMSO) were added. The Petri plates were labeled at the back. These plates were incubated at 37°C in the incubator for 24 h. The diameter of the clear zones was measured around each well as a zone of inhibition (ZOI; mm). The analyses of each sample were in triplicate. The minimum inhibition concentration (MIC) of the plant extract and TpAgNPs was measured by the method of 96-well microplate reader.

#### 2.5.4 Antifungal Assay

The antifungal activity was analyzed against *C. parapsilosis* (ATCC 22019), *C. albicans* (ATCC 9002), and *C. albicans* clinical isolate using the agar well diffusion method (Magaldi et al., 2004). The plant extract and biosynthesized TpAgNPs were dissolved in DMSO. Fungal suspensions (inoculums) were made by adding fungi in sterile distilled water. The inoculums were spread on prepared sterile Sabouraud dextrose agar (SDA) plates, 6 mm wells were made, and 40 µl of plant extract and biosynthesized TpAgNPs (5 mg/ml) was poured into each well. The standard antifungal drug Fluconazole (0.5 mg/ml) was taken as positive control and DMSO as negative control. The plates were incubated at 37°C in an incubator for 24–48 h. The results were based on the inhibition zones visible after 24–48 h and the diameter of the ZOI was measured in millimeter. The antimicrobial activity was calculated by taking the mean ± SD of the ZOIs.

### 2.6 Chemical Characterization of Biosynthesized TpAgNPs

The biosynthesized TpAgNPs were analyzed and characterized for the establishment of their morphology, structure, and surface function (Zia et al., 2017). The reduction of silver ion (Ag^+^) into TpAgNPs (Ag^0^) was observed in methanolic solution by UV-VIS spectrometer (T80^+^ PG instrument, United Kingdom) at 200–800 nm wavelength range. FTIR analysis was employed to identify the promising role of phytoconstituents of the plant extract capped on the surfaces of the biosynthesized TpAgNPs. The samples were scanned and monitored in the spectral ranges of 400–4,000 cm^−1^ using Nicolet iS5 (FTIR) spectrophotometer. X-ray diffraction (XRD) was used for the examination of both the crystal and molecular arrangements. XRD was obtained from Rigaku Geiger flex with Cu Kα1 radiation for 2 h values, and the scanning angle was 0–70°. Scanning electron microscopy (SEM) is a surface examining method, highly accomplished in undertaking different sizes of the particles and nanomaterial shapes of the created particles at the nano and micro levels. The shape and size of the biosynthesized silver nanoparticles were analyzed by SEM Jeol Japan JSM-IT 100.

### 2.7 Statistical Analyses

The data were analyzed by SPSS software 17.0 (SPSS Inc., Chicago, IL, United States). Triplicate analyses were made, and data were articulated as average and standard deviation (±SD). One-way ANOVA was performed with Duncan’s multiple range test for the measurement of significant differences (*p* ≤ 0.05) among the groups.

## 3 Results and Discussion

### 3.1 Determination of Total Phenolic, Flavonoid, and Anthocyanin Contents of Plant Extract

The extract of *T. pallida* was obtained and analyzed for quantitative determination of reducing agents, namely, total phenolic (TPC), total flavonoid (TFC), and total anthocyanin contents (TAC). The TPC was determined by Folin–Ciocalteu assay and the results revealed that the extract contains a significant amount of TPC: 26.67 ± 0.3 mg GAE/g. The TFC and TAC were determined by colorimetric and pH-differential methods and the extract contains 10.54 ± 1.3 mg RE/g and 1.402 ± 0.1 mg C3G/g/g, respectively (Table 1). The amount of these compounds in the extract is based on the selection of solvent systems and extraction techniques. It is reported that a polar solvent system containing acidified pH is more favorable for the extraction of flavonoids and specifically anthocyanins (Kapasakalidis et al., 2006; Simerdova et al., 2021). The results of the present study showed a 10-time increased amount of phenolic and flavonoid contents due to the selection of solvent system compared with previously reported values of phenolic (153.1 mg GAE/100 g) and flavonoid (10.6 mg RE/100 g) contents (Tan et al., 2014; Huq et al., 2016) of *T. pallida*. The anthocyanin content determination from *T. pallida* is reported for the first time, as no previous study was found for anthocyanin determination. However, Jabli (2018) reported that the color of the plant extract is dependent on the pH of the extract solution. The purple color of *T. pallida* exhibited anthocyanin contents, and as they are pH dependent, the acidic pH justified their stability and color. Hence, the quantity of these phytochemicals in the extract is based on the selection of solvent systems, pH, and extraction techniques. Therefore, in extracting anthocyanin-rich phenolics from plant materials, an organic solvent, i.e., 80% methanol (1:4; H_2_O:CH_3_OH v/v with 0.5% FA), was used in combination with a weak acid, such as formic acid (Kapasakalidis et al., 2006). The results revealed that *T. pallida* extract contains a significantly high quantity of polyphenolic components, especially flavonoids and anthocyanins, acting as potent reducing and capping agents in the nanoparticle formation process (Prasad, 2014; Mikhailova, 2020). These biomolecules present in *T. pallida* extract led to the formation of stable silver nanoparticles.

**TABLE 1:** Total phenolic, flavonoid, and anthocyanin contents of plant extract.

S. No.	Phytochemical analysis	*T. pallida* extract
1	Total phenolic content (mg GAE/g DW)	26.66 ± 0.3
2	Total flavonoid content (mg RE/g DW)	10.54 ± 1.3
3	Total anthocyanin content (mg C3GE/g DW)	1.402 ± 0.1

### 3.2 Visual Observation and Production of Silver Nanoparticles

The formation of silver nanoparticles of *T. pallida* extract (TpAgNPs) was observed visually by color change during the reduction process. The reaction mixture of *T. pallida* extract and AgNO_3_ solution at 0 min was reddish and turned to dark-brown after 24 h at room temperature. This demonstrated that after 24 h of incubation, the color intensity remained consistent, showing that the particles were well disseminated in the solution and not substantially aggregated. Plant extracts containing certain phytochemicals/secondary metabolites were involved in the reduction of silver ion (Ag^+^) to silver metal (Ag^0^), resulting in the color shift (Prasad, 2014; Mikhailova, 2020). The findings demonstrated that plant extract contains a wide spectrum of phytochemicals, with polyphenolic components, such as flavonoids and anthocyanins, acting as potent reducing and capping agents in the nanoparticle formation process (Abbasi et al., 2019). These biomolecules present in *T. pallida* extract led to the formation of stable TpAgNPs.

### 3.3 Biological Assays of Plant Extract and Biosynthesized TpAgNPs

#### 3.3.1 Antioxidant Assay

Antioxidant study of biosynthesized TpAgNPs and *T. pallida* plant extract was evaluated by DPPH % free radical scavenging activity. The change in color of DPPH from violet to colorless in ethanol was observed at 517 nm. The DPPH activity of the nine samples of TpAgNPs and *T. pallida* extract is shown in Table 2. Ascorbic acid (vitamin C) was used as standard and its % scavenging activity was 81.7 ± 0.85 with an IC_50_ value of 1.52 ± 1.0 μg/ml. In comparison to plant extract, silver nanoparticles demonstrated considerable (*p* ≤ 0.05) radical scavenging activity. The maximum % scavenging activity was 77.2 ± 1.0% of TpAgNP6 (5:10) among all nine samples. Similarly, TpAgNP5, TpAgNP4, and TpAgNP1 were also moderately active with % scavenging activity of 69.1, 68.3, and 63%, respectively, while other samples of silver nanoparticles were not active. Furthermore, TpAgNP6 (5:10) showed significantly (*p* ≤ 0.05) lowest IC_50_ of 18.54 ± 1.5 μg/ml; however, the IC_50_ values of TpAgNP5, TpAgNP1, and the plant extract were high and ranged from 52.7 to 62.5 μg/ml (Table 2). The results revealed that TpAgNP6 nanoparticles showed significant antioxidant activity due to the presence of bioactive reductants on the large surface of AgNPs. The phenolics, flavonoids, and anthocyanins, presented great antioxidant potential (Heim et al., 2002; Ranilla et al., 2010) and hence the presence of these compounds on the surface of biosynthesized silver nanoparticles is primarily responsible for the increased % scavenging activity (Sreelekha et al., 2021). Correspondingly, the % scavenging activity of the DPPH assay showed that TpAgNPs were highly active in comparison to *T. pallida* extract for antioxidant potential. This is the first report of the % scavenging activity of silver nanoparticles synthesized from *T. pallida*. DPPH is a free radical method of electron transfer and is reduced in the existence of antioxidant biomolecules (Huang et al., 2005; Asraoui et al., 2021). Hence, the free radicals of DPPH paired up with the electrons of antioxidants (TpAgNPs) and DPPH gets reduced, with a decrease in intensity of color (Akintola et al., 2020; Sreelekha et al., 2021). The trend of % scavenging activity increased with increasing concentration of plant extract, observed from a previous study (Huq et al., 2016), and a similar trend was observed for the nine TpAgNPs samples in the current study. However, TpAgNP6 (5:10) showed significantly highest scavenging activity of 77.2 ± 1.0%, which was near to the standard ascorbic acid having 81.7 ± 0.85%. Similarly, comparing the IC_50_ values of all nanoparticle samples with the standard showed that TpAgNP6 exhibited significant antioxidant potential (Table 2). The results revealed that nanoparticles synthesized at a medium concentration (5 mM) were proven to exhibit strong activities when tested at three distinct metal concentrations (1, 5, and 10 mM). It has also been observed that metal concentration and increasing amount of plant extract in the reaction media can alter nanoparticle production, changing the size and shape of nanoparticles (Hussain et al., 2019).

**TABLE 2:** Antioxidant activity of plant extract and biosynthesized TpAgNPs of *T. pallida*.

Samples	Scavenging %					IC_50_ (µg/ml)
	200 (µg/ml)	100 (µg/ml)	50 (µg/ml)	25 (µg/ml)	12.5 (µg/ml)	
Ascorbic acid	81.7 ± 0.85	81.1 ± 1.1	79.8 ± 1.5	79.3 ± 1.1	76.0 ± 0.85	1.52 ± 1.0^a^
TP extract	45.1 ± 0.5	39.8 ± 1.0	33.9 ± 0.64	26.5 ± 0.87	25.2 ± 1.0	62.5 ± 0.5^e^
TpAgNP1	63.0 ± 0.85	57.0 ± 1.0	45.1 ± 1.63	33.8 ± 1.3	31.3 ± 0.75	56.77 ± 1.5^d^
TpAgNP2	50.6 ± 1.25	38.7 ± 0.74	24.6 ± 1.73	22.2 ± 0.79	20.3 ± 1.5	94.4 ± 1.6^g^
TpAgNP3	49.8 ± 0.57	30.9 ± 0.85	16.8 ± 1.0	11.8 ± 1.5	9.0 ± 0.5	141.36 ± 0.5^h^
TpAgNP4	68.3 ± 0.89	57.6 ± 1.59	39.9 ± 1.53	37.0 ± 1.1	29.3 ± 1.0	84.22 ± 1.9^f^
TpAgNP5	69.1 ± 1.50	60.7 ± 0.06	55.0 ± 1.73	40.8 ± 0.5	39.0 ± 1.1	52.72 ± 1.05^c^
TpAgNP6	77.2 ± 1.0	74.2 ± 0.57	66.7 ± 1.5	57.6 ± 1.2	46.3 ± 0.79	18.54 ± 1.5^b^
TpAgNP7	56.0 ± 0.57	39.1 ± 0.89	29.2 ± 1.25	24.0 ± 0.85	9.9 ± 0.5	486.72 ± 1.25^j^
TpAgNP8	50.0 ± 0.5	37.5 ± 0.79	25.2 ± 0.57	14.2 ± 1.25	11.2 ± 0.75	85.04 ± 0.5^f^
TpAgNP9	53.5 ± 1.0	35.6 ± 0.74	24.9 ± 0.5	14.5 ± 1.1	13.3 ± 0.57	196.6 ± 1.1^i^

#### 3.3.2 Cytotoxicity Assay

**FIGURE 1:**
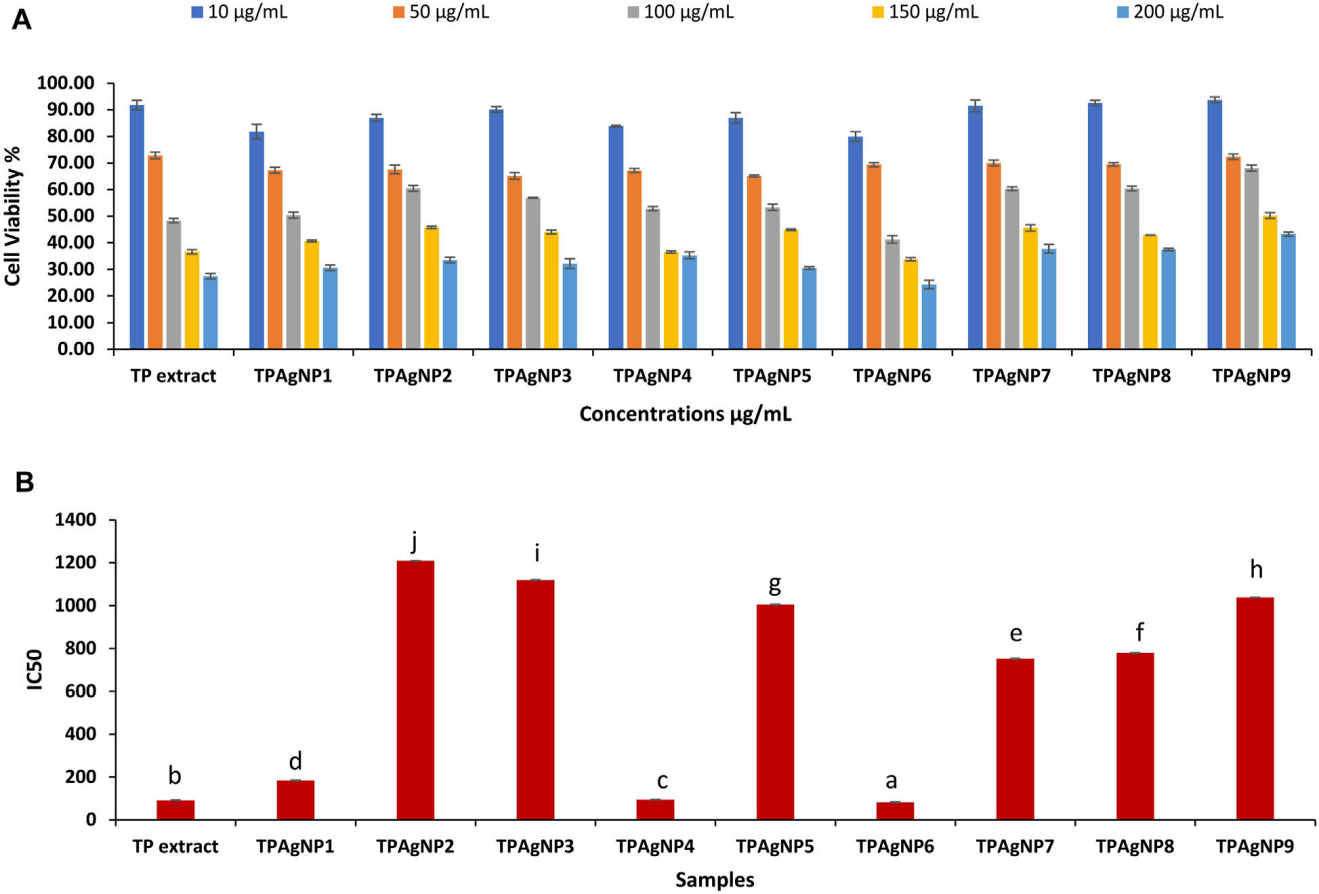
Result of cytotoxicity assay of plant extract and TpAgNPs of *T. pallida* **(A)** and IC_50_ values of extract and nanoparticles **(B)**.

The anticancer potential of *T. pallida* extract and its silver nanoparticles was determined using the MTT test. *T. pallida* extract exhibited good cytotoxicity with an IC_50_ value of 90.59 ± 1.6 μg/ml and cell viability % of 27.4 ± 1.05 (Supplementary Figure S1). However, in the nine samples of silver nanoparticles, TpAgNP6 indicated significantly (*p* ≤ 0.05) lowest IC_50_ value of 81.5 ± 1.9 μg/ml with cell viability % of 24.3 ± 1.62, followed by TpAgNP4 with a value of 35.3 ± 1.25 with IC_50_ of 93.7 ± 0.5 μg/ml (Figure 1). The methanolic extract and both TpAgNP6 and TpAgNP4 indicated a concentration-dependent decrease in cell viability %. The rest of the TpAgNP samples showed the highest IC_50_ values (752.5 ± 1.5 to 1,209.0 ± 2.0 μg/ml) and lower cytotoxicity compared to *T. pallida* extract. The results revealed that all samples showed a concentration-dependent decrease in cell viability (%). The decreased trend of cell viability (%) on increasing concentration of the sample follows the previous report. The results also indicated that the extract showed a potent effect with an IC_50_ value of 90.59 ± 1.6 μg/ml. This is the first report of the cytotoxic and anticancer potential of *T. pallida* extract and its biosynthesized silver nanoparticles (Supplementary Figure S1). These findings are comparable to the previous reports that both plant extract and biosynthesized silver nanoparticles worked effectively (Jeyaraj et al., 2013; Tariq et al., 2022). The biosynthesized TpAgNPs were also highly active; however, TpAgNP6 (5:10) sample was significantly (*p* ≤ 0.05) more active with an IC_50_ value of 81.5 ± 1.9 μg/ml in inhibiting cellular growth in comparison to all other nanoparticle samples and plant extract. It is therefore found that 5 mM metal ion concentration with 10 mg/ml of plant extract has a strong effect on cytotoxic activity. Interestingly, similar IC_50_ values were determined against a variety of cell lines as reported previously (Jeyaraj et al., 2013; Mohanta et al., 2017; Hemlata et al., 2020; Yan et al., 2020). The considerable antiproliferative effect of biosynthesized silver nanoparticles was demonstrated in this work. However, this activity might be due to the synergetic impact of nanosized silver and bioactive phytocompounds adhering to the nanoparticles’ surfaces. The cytotoxicity of silver nanoparticles is well known and is dependent on their size and the kind of cell (Park et al., 2011). Several physical features of silver nanoparticles, such as shape, surface area, and thickness, have been identified to influence their chemotherapeutic effects. Silver nanoparticles of small size have been found to migrate from the cell membrane and be removed from tumor cells at the current scale. In larger sizes, the potential is greatly decreased (Suman et al., 2013). Silver nanoparticles made from medicinal plant extracts have a high cytotoxic potential against cancer cells. Researchers discovered that antioxidant materials made from ethnomedicinal plants, such as silver nanoparticles, diminish tumor volume by eliminating free radicals and inhibiting the proliferation of all malignant cells (Sangami and Manu, 2017; Radini et al., 2018).

#### 3.3.3 Antibacterial Assay

The *T. pallida* plant extract and nine samples of biosynthesized silver nanoparticles were analyzed against Gram-negative (*P. aeruginosa*) and Gram-positive (*S. aureus,* methicillin-resistant *S. aureus*) bacteria. The antibacterial activity of all TpAgNPs (1–9), plant extract, positive control, and negative control is shown in Table 3. Activity comparison of all samples indicated that TpAgNP6 (5:10) was found to have a statistically significant (*p* ≤ 0.05) impact, with maximum ZOI of 28.3 ± 1.25, 27.3 ± 0.47, and 20.3 ± 0.47 mm against *P. aeruginosa*, *S. aureus*, and MRSA, respectively (Supplementary Figure S2). TpAgNP9 and TpAgNP7 were also significantly active against *S. aureus* (34.00 ± 0.82, 25.33 ± 1.25 mm, respectively) and MRSA (16.67 ± 1.25, 17.67 ± 0.47 mm, respectively); however, their activity was less against *P. aeruginosa* (8.33 ± 0.82, 16.00 ± 1.25 mm, respectively). Similarly, the TpAgNP4 sample was active against *S. aureus* (26.00 ± 0.82 mm) while less active against MRSA (12.67 ± 0.94 mm) and *P. aeruginosa* (20.33 ± 1.69 mm). Besides this, other TpAgNPs were moderately active. Therefore, TpAgNP6 (5:10) was selected for further study and its MIC value was obtained as 64, 128, and 128 μg/ml against *P. aeruginosa*, *S. aureus*, and MRSA, respectively (Table 4). The MIC results showed that TpAgNPs were most potent against Gram-negative bacteria. The antibacterial results also revealed that silver nanoparticles had higher activity than the plant extract due to their larger surface area, which allows for greater interaction with the bacteria’s cell surfaces. These findings were compared to prior research that found silver nanoparticles to have potent antibacterial properties (Pal et al., 2007; Al-Otibi et al., 2020; Bawazeer et al., 2021). In the present study, we reported the antibacterial activity of TpAgNPs against methicillin-resistant *S. aureus* clinical isolate and *P. aeruginosa* for the first time. It was reported that silver nanoparticles interact with bacterial cell walls and membranes and subsequently disrupt the cell organelles (Dakal et al., 2016), which led to DNA damage (Reidy et al., 2013; Duran et al., 2016). However, it was found in this study that TpAgNPs (5:10) synthesized at 5 mM of AgNO_3_ with a high concentration of plant extract exhibited strong activities. It was also reported that the quantity of plant extract and metal concentration in the reaction influence the process of nanoparticle production, which may lead to change in their sizes and shapes (Pal et al., 2007; de Melo et al., 2020). The medicinal plants have their therapeutic properties. In addition, the formation of silver nanoparticles predominates their persistent antibacterial effect and is sometimes superior to antibiotics (Ruiz-Baltazar et al., 2017).

**TABLE 3:** Zone of inhibition of plant extract and TpAgNPs of *T. pallida* against different bacterial strains.

Samples	Zone of inhibition (mm)		
	*P. aeruginosa*	*S. aureus*	*MRSA*
Tp extract	13.00 ± 0.82^kl^	7.33 ± 0.47^p^	8.00 ± 0.82^op^
TpAgNP1	11.00 ± 0.82^mn^	22.00 ± 0.82^gh^	12.33 ± 0.47^klm^
TpAgNP2	12.00 ± 00^lm^	24.33 ± 1.70^ef^	13.33 ± 0.47^kl^
TpAgNP3	15.67 ± 0.94^j^	23.67 ± 1.25^fg^	13.00 ± 0.82^kl^
TpAgNP4	20.33 ± 0.47^h^	26.00 ± 0.82^de^	12.67 ± 0.94^klm^
TpAgNP5	17.33 ± 1.69^ij^	22.00 ± 0.82^gh^	12.00 ± 0.82^lm^
TpAgNP6	28.33 ± 1.25^c^	27.33 ± 0.47^cd^	20.33 ± 0.47^h^
TpAgNP7	16.00 ± 0.82^ij^	25.33 ± 1.25^ef^	17.67 ± 0.47^i^
TpAgNP8	9.67 ± 1.25^no^	20.33 ± 0.47^h^	14.00 ± 0.82^k^
TpAgNP9	8.33 ± 0.47^op^	34.00 ± 0.82^b^	16.67 ± 1.25^ij^
DMSO	0.00 ± 0.00^q^	0.00 ± 0.00^q^	0.00 ± 0.00^q^
Ciprofloxacin	36.00 ± 0.82^a^	35.66 ± 0.47^a^	20.66 ± 0.47^h^

Tp, T. pallida; AgNP, silver nanoparticles; MRSA, methicillin-resistant S. aureus clinical isolate. Data represent mean ± standard deviation of triplicate.

**TABLE 4:** MIC of plant extract and TpAgNPs of *T. pallida* against different bacterial and fungal strains.

Samples	MIC (µg/ml)					
	*P. aeruginosa*	*S. aureus*	*MRSA*	*C. parapsilosis*	*C. albicans*	*C. albicans* ^*1*^
Tp extract	1,024	1,024	1,024	1,024	1,024	1,024
TpAgNP1	1,024	1,024	1,024	1,024	1,024	1,024
TpAgNP2	1,024	1,024	1,024	1,024	1,024	1,024
TpAgNP3	1,024	1,024	1,024	1,024	1,024	1,024
TpAgNP4	1,024	512	1,024	1,024	1,024	1,024
TpAgNP5	1,024	1,024	1,024	1,024	1,024	1,024
TpAgNP6	64	128	128	128	512	512
TpAgNP7	1,024	512	512	1,024	1,024	1,024
TpAgNP8	1,024	1,024	1,024	1,024	1,024	1,024
TpAgNP9	1,024	64	512	1,024	1,024	1,024

Tp, T. pallida; AgNP, silver nanoparticles; MRSA, methicillin-resistant S. aureus clinical isolate; 1, C. albicans clinical isolate.

#### 3.3.4 Antifungal Assay

The antifungal activity of *T. pallida* extract and the nine biosynthesized TpAgNP samples was tested against *C. parapsilosis*, *C. albicans*, and *C. albicans* clinical isolate (Table 5). All the biosynthesized TpAgNP samples showed significant antifungal activity while the plant extract was moderately active (Supplementary Figure S2). Among the nine samples of TpAgNPs, TpAgNP6 (5:10) showed statistically significant (*p* ≤ 0.05) antifungal activity, with maximum ZOIs of 23.00 ± 0.82, 22.67 ± 0.47, and 13.67 ± 0.94 mm against *C. parapsilosis*, *C. albicans,* and *C. albicans* clinical isolate, respectively. However, the other samples of TpAgNPs were active but their activity was less or none against the three *Candida* species. The antimicrobial activity of silver nanoparticles is attributed to several factors including their size, shape, surface area, surface charge, concentration of nanoparticles, duration of exposure, and species sensitivity of pathogens (Ghojavand et al., 2020). Although the mechanism of the fungicidal effect is not completely known, it has been suggested that silver nanoparticles impede budding by generating holes in the fungal cell membrane, which can result in cell death (Alwhibi et al., 2021; Lu et al., 2021; Paul and Roychoudhury, 2021).

**TABLE 5:** Zone of inhibition of plant extract and TpAgNPs of *T. pallida* against fungal strains.

Samples	Zone of inhibition (mm)		
	*C. parapsilosis*	*C. albicans*	*C. albicans* ^*1*^
Tp extract	10.50 ± 0.4^1hi^	9.00 ± 0.82^jkl^	8.33 ± 0.47^klm^
TpAgNP1	0.00 ± 0.00^p^	10.33 ± 0.47^hij^	0.00 ± 0.00^p^
TpAgNP2	0.00 ± 0.00^p^	7.33 ± 0.47^mno^	7.00 ± 0.82^o^
TpAgNP3	8.00 ± 0.82^lmn^	18.00 ± 0.82^e^	8.33 ± 1.25^klm^
TpAgNP4	9.50 ± 0.41^ijk^	15.67 ± 0.47^f^	7.67 ± 0.47^lmno^
TpAgNP5	16.20 ± 0.62^f^	13.67 ± 0.47^g^	8.00 ± 1.41^lmn^
TpAgNP6	23.00 ± 0.82^c^	22.67 ± 0.47^c^	13.67 ± 0.94^g^
TpAgNP7	6.67 ± 0.47^o^	11.33 ± 0.47^h^	0.00 ± 0.00^p^
TpAgNP8	6.33 ± 1.25^o^	10.00 ± 0.82^hij^	11.00 ± 0.82^h^
TpAgNP9	11.17 ± 0.62^h^	11.00 ± 0.82^h^	10.67 ± 0.47^hi^
DMSO	0.00 ± 0.00^p^	0.00 ± 0.00^p^	0.00 ± 0.00^p^
Fluconazole	29.00 ± 0.41^a^	27.47 ± 0.82^b^	20.47 ± 0.82^d^

Tp, T. pallida; AgNP, silver nanoparticles; 1, C. albicans clinical isolate. Data represent mean ± standard deviation of triplicate.

### 3.4 Chemical Characterization of Biosynthesized TpAgNPs

#### 3.4.1 UV–Visible Spectroscopy Analysis

**FIGURE 2:**
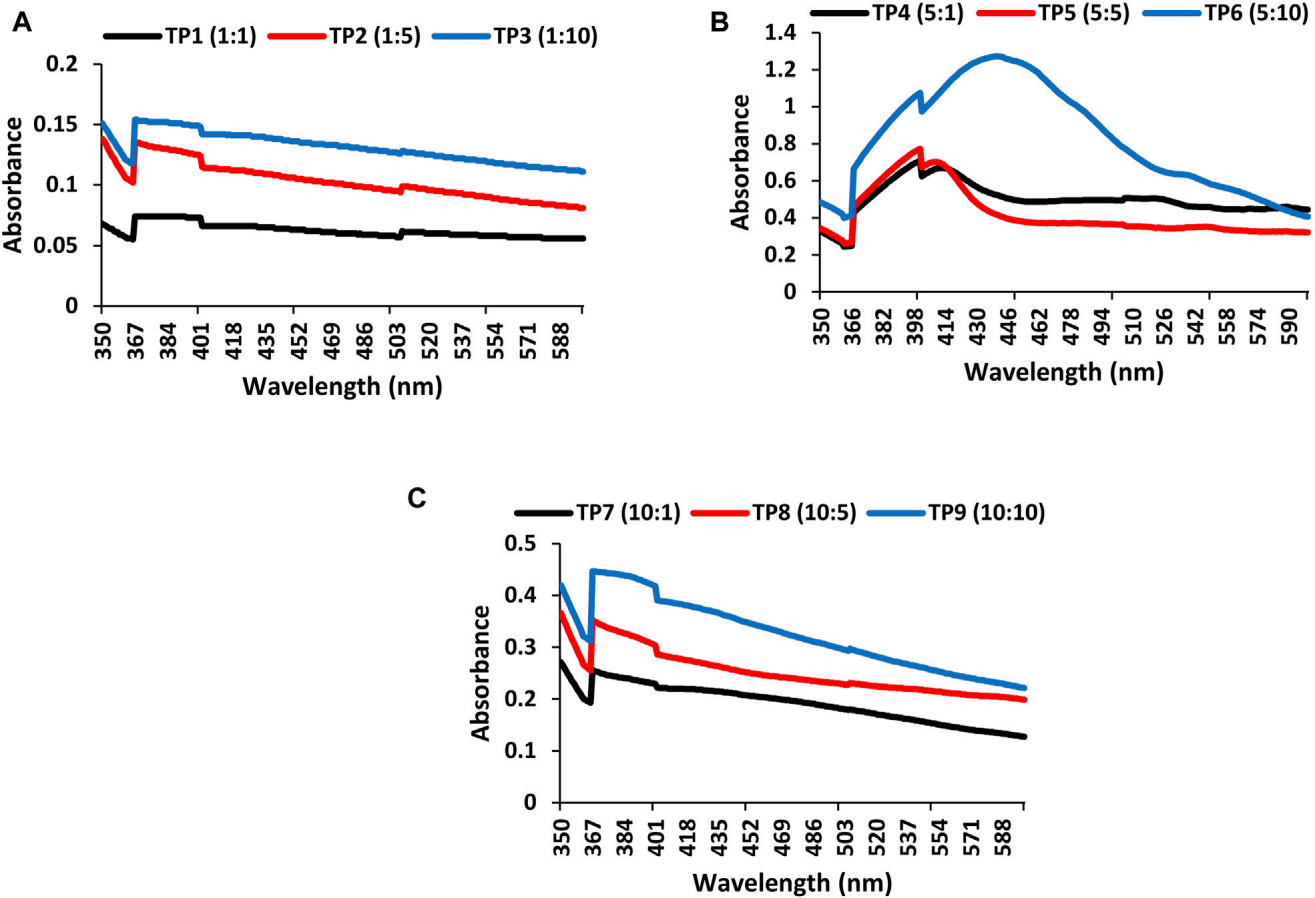
UV–Vis spectra of TpAgNPs synthesized by different concentrations of AgNO_3_ and plant extract: **(A)** *Tradescantia pallida* (TP) silver nanoparticles synthesized with 1 mM AgNO_3_ and 1, 5, and 10 mg ml^−1^ of plant extract (TP1, TP2, and TP3), **(B)** *Tradescantia pallida* (TP) silver nanoparticles synthesized with 5 mM AgNO_3_ and 1, 5, and 10 mg ml^−1^ of plant extract (TP3, TP4, and TP5), **(C)** *T. pallida* (TP) silver nanoparticles synthesized with 10 mM AgNO_3_ and 1, 5, and 10 mg ml^−1^ of plant extract (TP7, TP8, and TP9).

The formation of TpAgNPs in colloidal solution was confirmed by UV–visible absorption spectra. The absorption spectra of the nine different samples of TpAgNPs with increasing concentrations of *T. pallida* plant extract of 1, 5, and 10 mg ml^−1^ and 1, 5, and 10 mM AgNO_3_ are presented in Figure 2. The peak absorbance for TpAgNPs was recorded in the 350–600 nm wavelength range (Figure 2). UV–Vis spectroscopy is a widely used method for determining the stability of metal nanoparticles, such as AgNPs (Jyoti et al., 2016). The surface plasmon resonance (SPR) band of silver nanoparticles appeared at a range of 390–441 nm and confirms the formation of TpAgNPs (Figure 2). Because of the existence of free electrons in silver metal, nanoparticles with visible light give rise to the SPR band, which shows significant absorption of electromagnetic waves (Serra et al., 2009; Baliyan et al., 2017). The increase in the quantity of plant extract shows increase in band intensity (Figure 2). The quality and quantity of plant extract have great potential for stabilizing components to act as reductants (Groning et al., 2001; Ronavari et al., 2021). The concentration of plant extract and AgNO_3_ plays an important role in silver nanoparticle formation, particularly particle morphology and size (de Melo et al., 2020; Ronavari et al., 2021). At a low concentration of AgNO_3_ (1 mM) with an increasing concentration of plant extract, the band for TpAgNPs existed in the range of 393–402 nm (Figure 1A). At medium AgNO_3_ (5 mM) concentration with plant extract, it was 401–441 nm in range (Figure 1B), while at higher concentration of AgNO_3_ (10 mM), the availability of plant extract as a bio-stabilizing agent decreased. Therefore, the medium concentration (5:10) was selected for further characterization. The ratio of *T. pallida* extract to AgNO_3_ to produce TpAgNPs was optimized for the first time. Our results also revealed that nanoparticles (TpAgNP6) at a medium concentration ratio (5:10) were biologically more active for antioxidant, anticancer, antibacterial, and antifungal activities.

#### 3.4.2 Fourier Transform Infrared Spectroscopy Analysis

**FIGURE 3:**
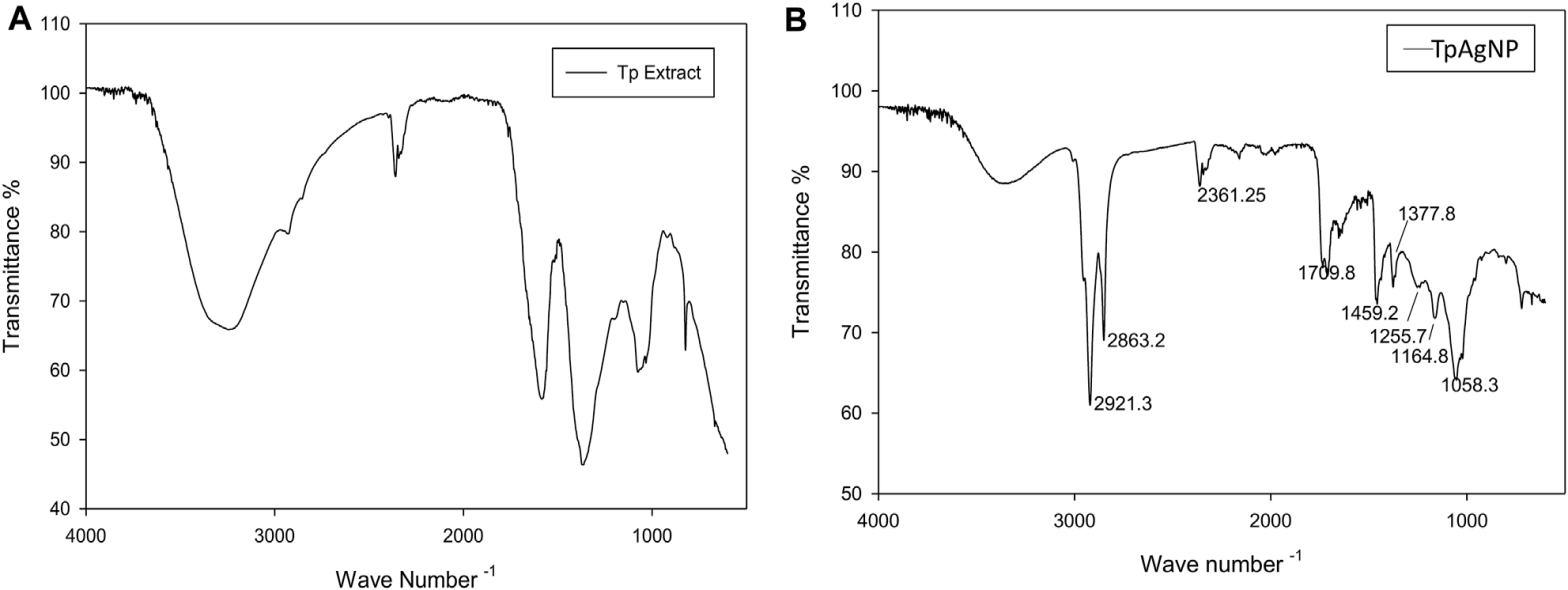
FTIR spectral analyses of **(A)** plant extract and **(B)** synthesized TpAgNPs of *T. pallida*.

FTIR analyses were made to determine the potential biomolecules in *T. pallida* plant extract and TpAgNPs. This characterization technique is used to analyze different functional groups and biomolecules involved in the stabilization of silver nanoparticles (Kiefer et al., 2015; Chartarrayawadee et al., 2020). The absorption peaks of spectra were analyzed from 4,000 to 600 cm^−1^. The absorption peaks of plant extract were observed at 823, 1,082, 1,374, 1,584, 1,761, 2,352, 2,362, 2,873, 2,935, and 3,292 cm^−1^ (Figure 3A). Similarly, the spectra of TpAgNPs showed major peaks at 721, 1,058, 1,165, 1,256, 1,378, 1,459, 1,710, 2,361, 2,853, 2,922, and 3,366 cm^−1^. Additionally some minor peaks were also observed (Figure 3B). The broad peaks at 3,366 and 3,292 cm^−1^ showed the existence of O─H (H-bond stretching) vibration of phenolic compounds (Keshari et al., 2020; Patle et al., 2020). The peaks at 2,935, 2,922, 2,873, 2,853, 2,361, 2,362, and 2,352 cm^−1^ denoted the C─H stretching vibration of -CH_3_ groups, aldehydes H─C═O, nitriles C**≡**N, and alkynes C≡C groups, respectively (Keshari et al., 2020). The corresponding peaks at 1,761, 1,710, 1,584, 1,459, 1,378, 1,374, and 1,256–1,058 cm^−1^ were due to stretching vibration of alkenes C═C in aromatic rings, carbonyl groups C═O of flavonoids, and amide groups N–H of the proteins (Jyoti et al., 2016; Katta and Dubey, 2021). However, the remaining peaks at 721 and 823 cm^−1^ were representing the stretch vibration of alkyl halide (C─Cl) of anthocyanins (Abbasi et al., 2019). These results revealed that the stretching vibration peaks, reduction/capping agents, and stabilization of synthesized silver nanoparticles are mainly due to the flavonoids, specifically anthocyanins, as important phytochemicals present in the *T. pallida* extract (Dash et al., 2020).

#### 3.4.3 X-Ray Diffraction Analysis

**FIGURE 4:**
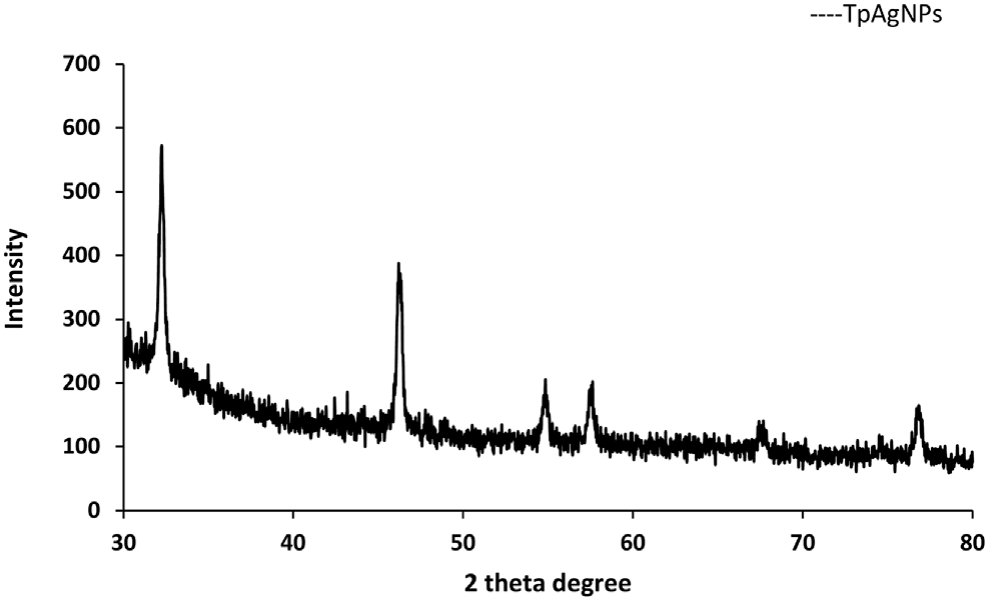
X-ray diffraction analysis of biosynthesized TpAgNPs from *T. pallida*.

The XRD analysis was used to investigate the crystalline structure of silver nanoparticles (Figure 4). The pattern of four diffraction peaks of TpAgNPs originated in Bragg’s angle ranged at 32.2°, 46.3°, 67.4°, and 77.2° corresponding to (111), (200), (220), and (311), respectively. The range of Bragg’s angle (2*h* ꞊ 35–80) depicts the face-centered cubic form of silver phases as noted by the XRD pattern previously (Katta and Dubey, 2021). The Scherrer equation calculated the crystal size of TpAgNPs as 29.7 nm (Rabiei et al., 2020). The peak pattern of XRD substantially impacted the size of the particle (Aziz et al., 2019; Rabiei et al., 2020). The existence of some phytochemical/organic substances in the plant extract covering the surface of the produced silver nanoparticles and stabilizing them might explain the unexplained peaks detected in the XRD spectra (Krishnaraj et al., 2010). Our findings corroborate those of Okaiyeto et al. (2021), who found that the XRD peaks of biosynthesized silver nanoparticles corresponded to cubic crystalline silver.

#### 3.4.4 Scanning Electron Microscopy

**FIGURE 5:**
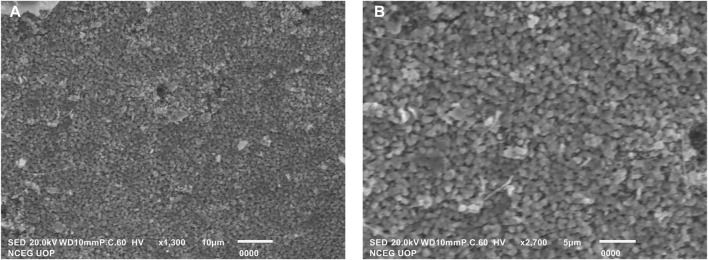
SEM images **(A)** and **(B)** spherical shape of biosynthesized TpAgNPs.

The appearance of biosynthesized TpAgNPs was examined and confirmed using SEM. Figure 5 shows the spherical form of silver nanoparticles as seen by SEM. These findings revealed that the capping of bioactive chemicals in *T. pallida* extract caused the production of nanoparticles. The shape of silver nanoparticles was recognized to cause significant changes in optical and electrical characteristics (Chen et al., 2004). Various studies on biosynthesized nanoparticles have revealed that the stability of silver nanoparticles is due to the reducing agents present in the plant extract, which is further responsible for the crystalline structure and spherical shape of nanoparticles. The presence of different reductants in the plant extract is responsible for the stabilization of silver nanoparticles and, as a result, for the crystalline structure and spherical form of silver nanoparticles, which has been thoroughly investigated in numerous biosynthesized nanoparticles. The surface area and size of silver nanoparticles are also responsible for their biological activity; smaller nanoparticles have a bigger surface area than larger ones. According to earlier research, spherical nanoparticles are more efficient than other structures of nanoparticles (Zhao et al., 2017).

## Conclusion

The current study was about the potential exploration of antioxidant, cytotoxic, and antimicrobial activities to synthesize TpAgNPs for the first-time using *T. pallida* extract. UV–Vis, FTIR, XRD, and SEM methods were used to confirm the composition and properties of the nanoparticles. Generally, therapeutic efficiency revealed concentration-dependent antioxidant and cytotoxic activities of TpAgNPs. Our findings show that green nanomaterial creation from *T. pallida* might be employed as a potential drug discovery tool.

## Data Availability

The original contributions presented in the study are included in the article/Supplementary Material; further inquiries can be directed to the corresponding authors.

## Supplementary Material

Supplementary Figure S1 Image of MTT test of *T. pallida* extract and synthesized silver nanoparticles (TpAgNP6) of rhabdomyosarcoma cells.

Supplementary Figure S2 Antimicrobial Activity of *T. pallida* extract and synthesized silver nanoparticles (TpAgNPs).
