# Supplementary material for: Antioxidant, Cytotoxic, and Antimicrobial Potential of Silver Nanoparticles Synthesized using Tradescantia pallida Extract
Source: Front Bioeng Biotechnol. 2022 Jul 18;10:907551. doi: 10.3389/fbioe.2022.907551 (PMC9340775; doi:10.3389/fbioe.2022.907551)
Supplement: Supplementary file 1 [file DataSheet1.PDF]

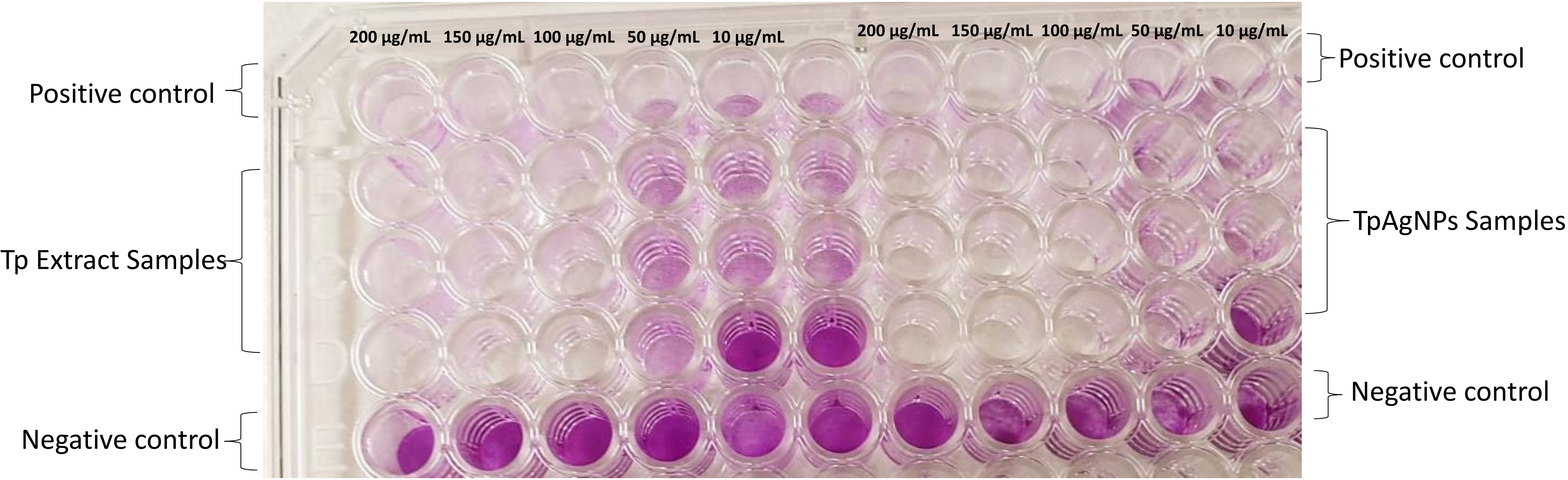

Supplementary Figure 1. Image of MTT test of *T. pallida* extract and synthesized silver nanoparticles (TpAgNP6) of rhabdomyosarcoma cells.

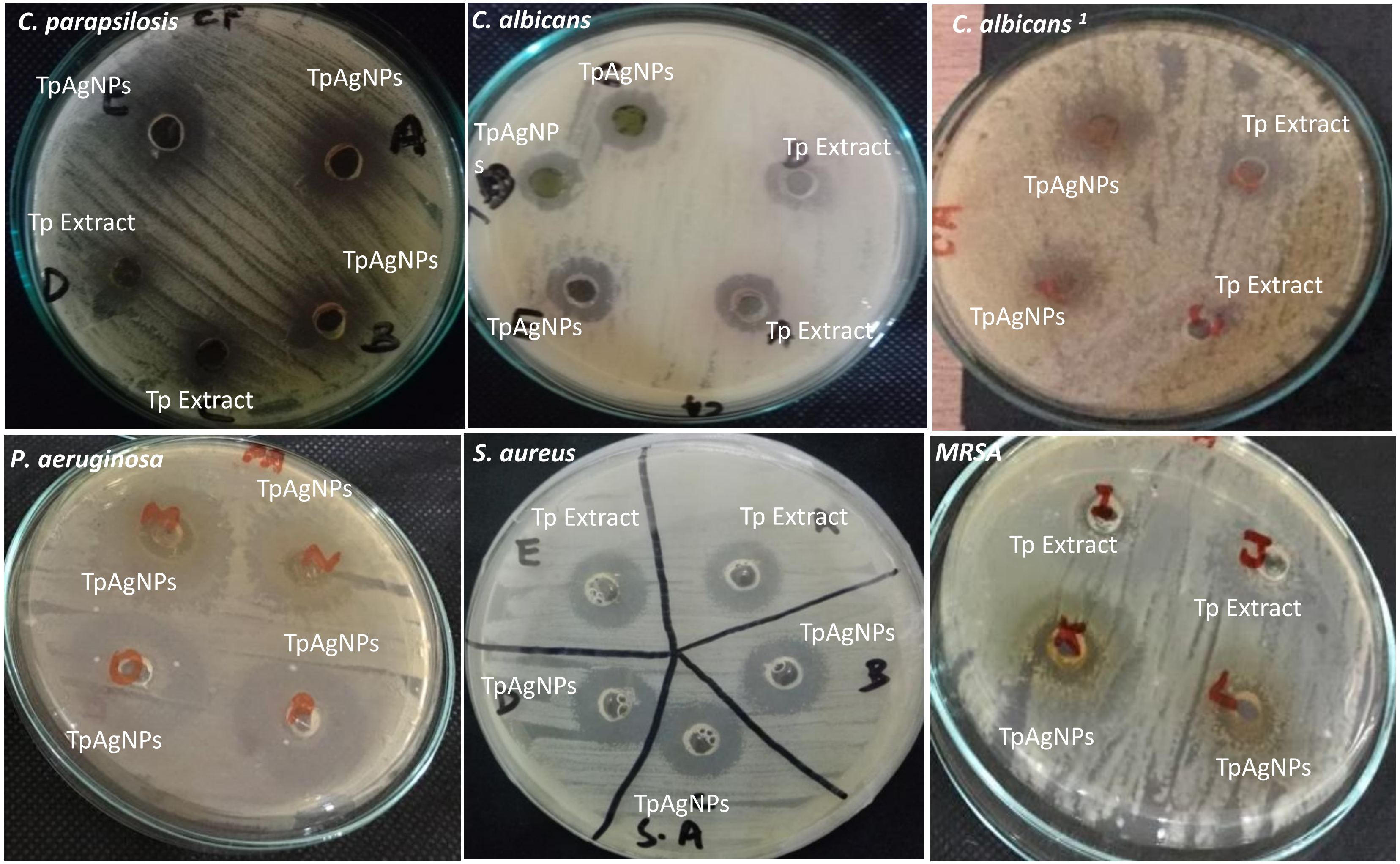

Supplementary Figure 2. Antimicrobial Activity of *T. pallida* extract and synthesized silver nanoparticles (TpAgNPs).
